# SCAR32: Functional characterization and expansion of the clinical‐genetic spectrum

**DOI:** 10.1002/acn3.52094

**Published:** 2024-06-05

**Authors:** Valentina Naef, Maria Lieto, Sara Satolli, Rosa De Micco, Martina Troisi, Rosa Pasquariello, Stefano Doccini, Flavia Privitera, Alessandro Filla, Alessandro Tessitore, Filippo Maria Santorelli

**Affiliations:** ^1^ Department Neurobiology and Molecular Medicine IRCCS Fondazione Stella Maris Pisa 56128 Italy; ^2^ Department of Neurology and Stroke Unit Ospedale del Mare Hospital Naples Italy; ^3^ Department of Advanced Medical and Surgical Sciences University of Campania “Luigi Vanvitelli” Naples Italy; ^4^ Department of Neurosciences Reproductive and Odontostomatological Sciences Federico II University Naples Italy

## Abstract

**Objective:**

Biallelic mutations in *PRDX3* have been linked to autosomal recessive spinocerebellar ataxia type 32. In this study, which aims to contribute to the growing body of knowledge on this rare disease, we identified two unrelated patients with mutations in *PRDX3.* We explored the impact of *PRDX3* mutation in patient skin fibroblasts and the role of the gene in neurodevelopment.

**Methods:**

We performed trio exome sequencing that identified mutations in *PRDX3* in two unrelated patients. We also performed functional studies in patient skin fibroblasts and generated a “crispant” zebrafish (*Danio rerio*) model to investigate the role of the gene during nervous system development.

**Results:**

Our study reports two additional patients. Patient 1 is a 19‐year‐old male who showed a novel homozygous c.525_535delGTTAGAAGGTT (p. Leu176TrpfsTer11) mutation as the genetic cause of cerebellar ataxia. Patient 2 is a 20‐year‐old male who was found to present the known c.425C>G/p. Ala142Gly variant in compound heterozygosity with the p. Leu176TrpfsTer11 one. While the fibroblast model failed to recapitulate the pathological features associated with *PRDX3* loss of function, our functional characterization of the *prdx3* zebrafish model revealed motor defects, increased susceptibility to reactive oxygen species‐triggered apoptosis, and an impaired oxygen consumption rate.

**Conclusions:**

We identified a new variant, thereby expanding the genetic spectrum of *PRDX3*‐related disease. We developed a novel zebrafish model to investigate the consequences of *prdx3* depletion on neurodevelopment and thus offered a potential new tool for identifying new treatment opportunities.

## Background

The term hereditary ataxia (HA) refers to a highly heterogeneous group of disorders phenotypically characterized by progressive damage to the cerebellum and/or its associated afferent tracts.^1^, ^2^ Collectively, these disorders have an estimated prevalence of 26/100,000 children.^3^ Although more than 200 genes have been associated with different types of HA, many patients remain undiagnosed, particularly if they are simplex cases with a silent family history. Among the key molecular “hubs” underlying HA, oxidative stress and mitochondrial dysfunction emerge as common themes. When the balance between cellular oxidants and antioxidants is shifted in favor of the former, the cells tend to overproduce reactive oxygen species (ROS). This leads to mitochondrial damage, impaired oxidative phosphorylation, altered membrane permeability, and alterations of Ca^2+^ homeostasis.^4^ All these changes amplify motor and cognitive dysfunction and accelerate the development of neurodegeneration.^5^, ^6^ The role of deregulated expression or activity of antioxidant enzymes in neurodegeneration has recently received considerable attention. Peroxiredoxins (PRDXs) are members of a conserved peroxidase family, and they maintain intracellular ROS homeostasis.^7^ In mammals, six distinct PRDXs have been identified (PRDX1–PRDX6) and all contain conserved reactive cysteine residues in the active site essential for their antioxidant activity.^8^ An increasing number of studies suggests that these enzymes may be involved in the process of neurodegeneration.^7^ Among the PRDX proteins, PRDX3, which appears to be localized only in neuronal mitochondria,^8^, ^9^ is considered a master regulator of mitochondrial quality control, regulating both ROS removal and mitophagy in mitochondria.^10^ Recently, mutations in *PRDX3* have been linked to autosomal recessive spinocerebellar ataxia type 32 [SCAR32; MIM #619862].^11^, ^12^, ^13^, ^14^ Since the first description of the *PRDX3‐*associated phenotype,^12^ 15 individuals harboring pathogenic variants have been described with onset ages ranging from infancy to adulthood.^12^, ^13^, ^14^, ^15^, ^16^ In vitro knockdown of *PRDX3* results in reduced levels of glutathione peroxidase activity and increased susceptibility to apoptosis triggered by ROS.^11^ The fruit fly model of the disease showed a deficit in locomotor activity and reduced survival times upon exposure to oxidative stress,^11^ whereas a knockout murine model (*Prdx3* ^*−/−*^) displayed reduced mitochondrial DNA content and ATP production, as well as impaired glucose tolerance and insulin resistance,^17^ suggesting that the gene product plays an important role in multiple processes. The early manifestations of impaired motricity and cognition suggest that PRDX3 plays a role in neurodevelopment. However, to date, no model for studying this aspect has been available. *Danio rerio* (the zebrafish) is a valuable organism for studying several human diseases including the different forms of HA,^18^, ^19^, ^20^ but until now there has existed no zebrafish model for investigating *prdx3* functions during neurodevelopment. Here, we describe a new biallelic pathogenic variant in *PRDX3* occurring in two unrelated boys who presented an early onset, slowly progressive cerebellar syndrome, external ophthalmoplegia, global hypokinesia, and behavioral changes. We further explored the impact of the gene mutation in patient dermal fibroblasts, as well as the effects of depletion of the gene in zebrafish, in the latter case by designing the “crispant” model that displays stable gene downregulation^21^, ^22^ without off‐target or toxic effects on embryonic development that could mask the phenotype. Our findings provide additional evidence supporting the involvement of PRDX3 in neurodevelopment.

## Methods

### Cellular model

The cellular study methods are reported as Supplementary Material.

#### prdx3 zebrafish model

Downregulation of gene function in zebrafish was carried out using a zebrafish wild‐type strain (WT AB), adopting standard breeding conditions.^23^ Zebrafish handling and experimental procedures were performed under the supervision of the Animal Care and Use Committee of the IRCCS Stella Maris Foundation (Pisa, Italy), in compliance with European Directive No. 63 on the protection of animals used for research, dated September 22, 2010. Every effort was made to minimize both animal suffering and the number of animals needed to collect reliable scientific data. The zebrafish *prdx3* gene has seven coding exons and encodes a 256‐amino acid protein. Multiple alignments of human PRDX amino acid sequences were performed using Protein BLAST (https://blast.ncbi.nlm.nih.gov/ accessed on December 01, 2023) (see Figs. S1 and S2). This in silico analysis revealed high percentage of identity for all prdx protein family members among fish and human, indicating that peroxiredoxins are highly evolutionary conserved across different species. In particular, we found a high conservation of the prdx3 protein with 70% identity between the human (ENST00000298510.4) and zebrafish (ENSDART00000041700.9) sequences, suggesting that prdx3 can be considered the fish ortholog of the human counterpart with a conserved putative function (see Fig. S1).

We generated a *prdx3* knockdown in zebrafish injecting single‐guide RNA (sgRNAs) together with Cas9 protein in single‐cell stage embryos (100 pg of sgRNAs and 100 pg of Cas9).^22^ The single‐guide RNAs (sgRNAs) were chosen from the list of the five best targets identified by CHOPCHOP software (https://chopchop.cbu.uib.no/), all found to have zero off‐target effects, and to be complementary to *prdx3* in facilitating mRNA degradation. DNA was extracted from embryos at 24 hours postfertilization (hpf), and CRISPR/Cas9 editing efficacy was confirmed by Sanger sequencing. The toxic effect after sgRNA injection was calculated by counting dead and alive embryos at 48 hpf. Behavioral evaluations focused on coiling activity, touch‐evoked escape responses, and locomotor tracking, and were performed using previously described protocols.^22^, ^24^ Total RNA was extracted from 20 embryos, at different stages, using Quick RNA miniprep (Zymo Research, Irvine, CA, USA) according to the manufacturer's instructions. cDNA and qRT‐PCR were performed as described by Naef et al^25^ Relative expression levels of each gene were calculated using the 2^−∆∆Ct^ method.^26^ The results obtained in at least three independent experiments were normalized to the expression of the housekeeping gene, β‐actin (ENSDARG00000037746). To evaluate intracellular ROS production, the in vivo carboxy‐H2DCFDA fluorescent probe (#8206004, Abcam, Cambridge, MA) was used as reported by Naef et al^25^ Mitochondrial respiration was analyzed in wild‐type and *prdx3‐*deficient (“crispant”) larvae at 120 hpf using the XFe24 analyzer (Seahorse Bioscience, North Billerica, MA) as described by Rollwitz and Jastroch.^27^ Apoptotic cells from larvae at 24 hpf were detected by staining with acridine orange (#235474, Sigma‐Aldrich, St. Louis, MO) as described by Xia et al^28^ All data reported in the manuscript come from three or more independent experiments giving similar results. Data were analyzed by applying either parametric or nonparametric methods, depending on the distribution of the response variable in question, as shown by the Shapiro–Wilk test. Statistical analysis used GraphPad Prism 6 software; data were plotted as the mean ± standard error of the means. Statistical significance is reported as follows: *p ≤ 0.05, **p ≤ 0.01, ***p ≤ 0.001, or ****p ≤ 0.0001.

## Results

### Case description

Patient 1 is a 19‐year‐old male admitted to our neurology department with imbalance and clumsiness present since the age of 10 years, when he also started to manifest introversion, social isolation, and academic decline. With the exception of a mild language delay, his developmental milestones were reached on time. The family history was unremarkable for neurological disease. Neurological examination showed a wide‐based gait with positive Romberg's test, slurred speech, and global hypokinesia. Tandem gait was not possible. He also presented upper and lower limb dysmetria, slight upper limb postural tremor, and hyporeflexia. Saccades were limited in the horizontal and vertical planes and associated with gaze‐evoked nystagmus. No pyramidal signs were detected. His Scale for the Assessment and Rating of Ataxia (SARA) score was 7/40. Extensive biochemical and neurometabolic investigations were all negative. Motor and sensory conduction studies were also normal. Brain magnetic resonance imaging (MRI) showed diffuse cerebellar atrophy associated with T2‐weighted hyperintensity of the middle cerebellar peduncles. An extensive neuropsychological evaluation revealed global cognitive functioning within normal limits.

Patient 2 is 23‐year‐old male born at term to healthy, unrelated parents. The family history was remarkable due to the patient's elder brother having intellectual disability and absence epilepsy since the age of 5 and psychiatric manifestations since his late teens. The proband had delayed developmental milestones with a mild language delay and learning difficulties in childhood. In his early teens, he presented gait ataxia and exercise intolerance. Shortly afterward he began experiencing frequent falls and difficulty climbing stairs, and brain MRI showed cerebellar atrophy. When examined by us at age 20, Patient 2 showed clumsy gait and was found to have a mood disorder. He could walk unsupported but unsteadily and with a wide gait. Tandem gait was not possible. He also presented upper limb dysmetria, slow saccades, and dysarthria. His SARA score was 10/40. According to the caregiver, he had recently developed sluggish hypophonic speech, upper limb rigidity, and occasional difficulty swallowing both solids and liquids.

Having ruled out the most common forms of spinocerebellar ataxia (SCA) and autosomal recessive cerebellar ataxia (ARCA), we performed trio exome sequencing (ES), as reported elsewhere,^29^ in both of the cases. In Patient 1, ES revealed a homozygous variant (c.525_535delGTTAGAAGGTT/ p. Leu176TrpfsTer11) in *PRDX3* (NM_006793.5) located in the PRX_Tyrp2cys domain of the protein. The parents were healthy carriers of the gene variant not previously reported in gnomAD and classified as “Likely Pathogenic” (PVS1 – very strong and PM2 – moderate) according to the American College of Medical Genetics and Genomics (ACMG) guidelines. Patient 2 harbored the c.425C>G/p. Ala142Gly on the paternal allele in compound heterozygosity with the c.525_535delGTTAGAAGGTT on the maternal one. The c.425C>G variant is classified as “Uncertain significance” (PM2‐moderate, PP5‐supporting) according to ACMG guidelines with a low frequency in gnomAD database (<0.01%), and it predicts a deleterious effect on protein (CADD = 25.6, REVEL = 0.45 scores). This variant has been reported in literature (Rebelo et al. 2021).

A cultured skin fibroblast cell line was available only for Patient 1. RT‐PCR amplification study demonstrated low mRNA expression levels as compared to a normal control. Sanger sequencing of cDNA from skin cells showed the c.525_535delGTTAGAAGGTT mutation and also a weakly expressed transcript possibly skipping exon 6 (not shown). These data do not exclude the occurrence of a nonsense‐mediated mRNA decay (NMD) mechanism, that still remains a possibility (Rebelo et al. 2021).

**Figure 1:**
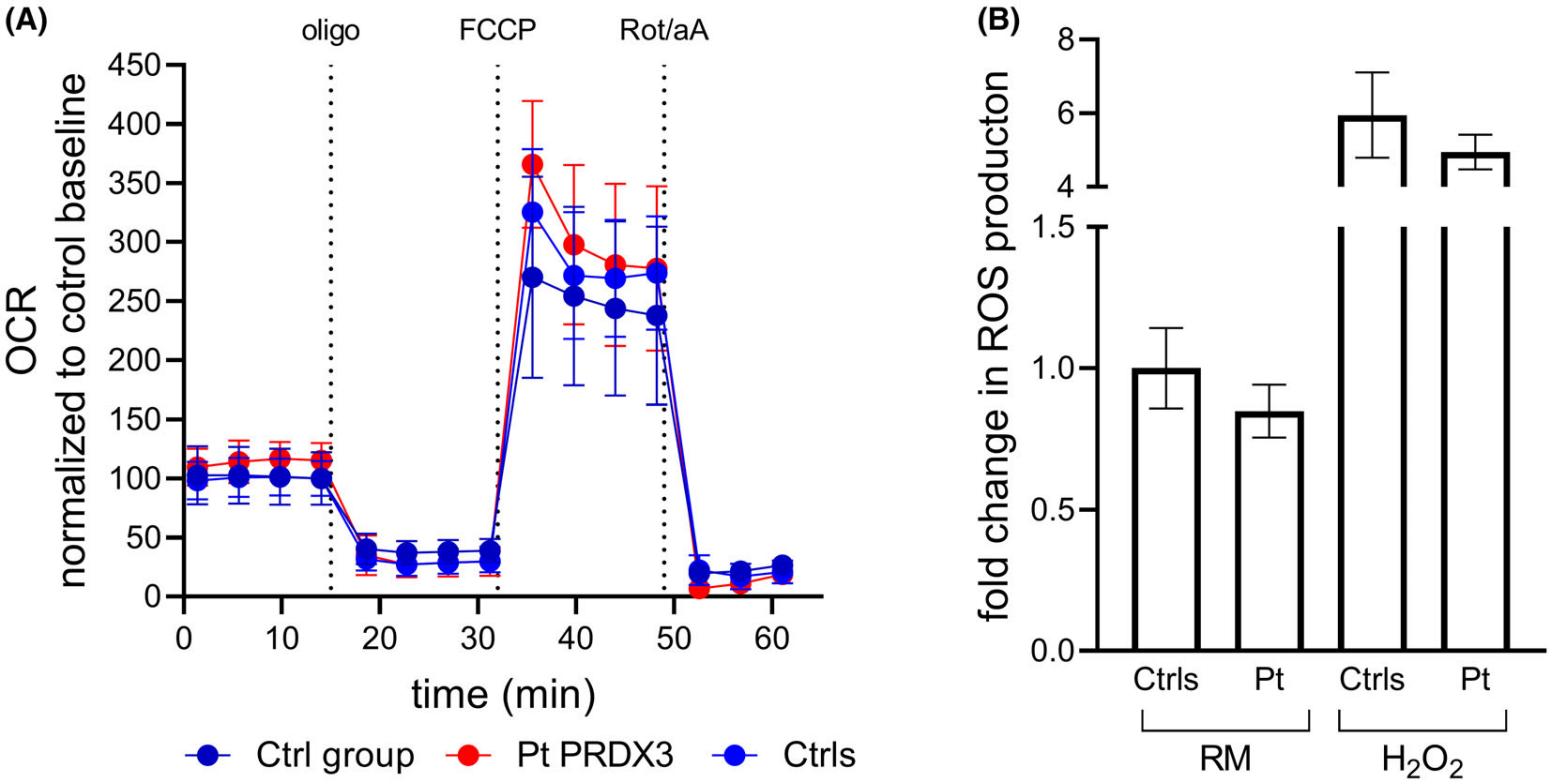
(A) Kinetic micro‐oxygraphy graph depicting mean ± SD of OCR values generated using the XFe24 analyzer (Seahorse Biosciences) under basal conditions and in response to the indicated drugs. Metabolic parameters obtained from OCR traces did not show significant changes in mitochondrial function between the patient and the controls. Data (Ctrls: five replicates from three different control cell lines; Pt PRDX3: 10 replicates from the patient cell line; Ctrl group: data (obtained from 31 previously tested control lines) were expressed as pmol O_2_/minute and normalized post assay to Hoechst 33342 intensity, as a function of the number of cells. Micro‐oxygraph traces were further normalized as percent of control baseline. Oligo: 2‐μM oligomycin; FCCP: 2‐μM carbonyl cyanide 4‐(trifluoromethoxy) phenylhydrazone; Rot/aA: 0.5/0.5‐μM rotenone/antimycin A. OCR, oxygen consumption rate. (B) Evaluation of susceptibility to oxidative stress in patient fibroblasts under both regular medium (RM) and stress conditions. Data represent mean ± SD of controls (*n* = 3) and the patient analyzed in technical triplicate. Statistical analysis performed by ordinary ANOVA test (one‐way ANOVA) did not show significant differences between patient and controls.

Biallelic *PRDX3* mutations have recently been described in SCAR32 (OMIM #619862) and associated with impaired oxidative stress defense and mitochondrial dysfunction. Functional analyses in cultured skin fibroblasts derived from Patient 1 did not display bioenergetic changes when compared with results obtained in healthy controls. Calculation of the key parameters of mitochondrial function, performed using the Seahorse XF Cell Mito Stress Test (Fig. 1), showed normal levels of basal respiration, ATP production, maximal respiration, and spare respiratory capacity, as well as normal proton leak and nonmitochondrial oxygen consumption. No significant changes in mitochondrial ROS levels were observed when ROS were measured using the H2DCFDA probe both in regular medium and in the presence of hydrogen peroxide to evaluate the cellular free radical scavenging capacity (Fig. 1). Our data suggest that the presumptive neuronal functions of PRDX3 are not seen in peripheral cell tissues.

#### In vivo studies in zebrafish

In zebrafish, six peroxiredoxins (prxd1‐6) have been identified.^30^ However, as far we know only prdx1 has fully been characterized in zebrafish sharing the 80% of similar identity with the human protein.^30^, ^31^ Therefore, we performed multi‐alignment analysis of the amino acid sequence of all prdx genes in zebrafish compared to human proteins and observed that all prdx members in zebrafish are the fish orthologs of the human counterpart (Figs. S1 and S2).

**Figure 2:**
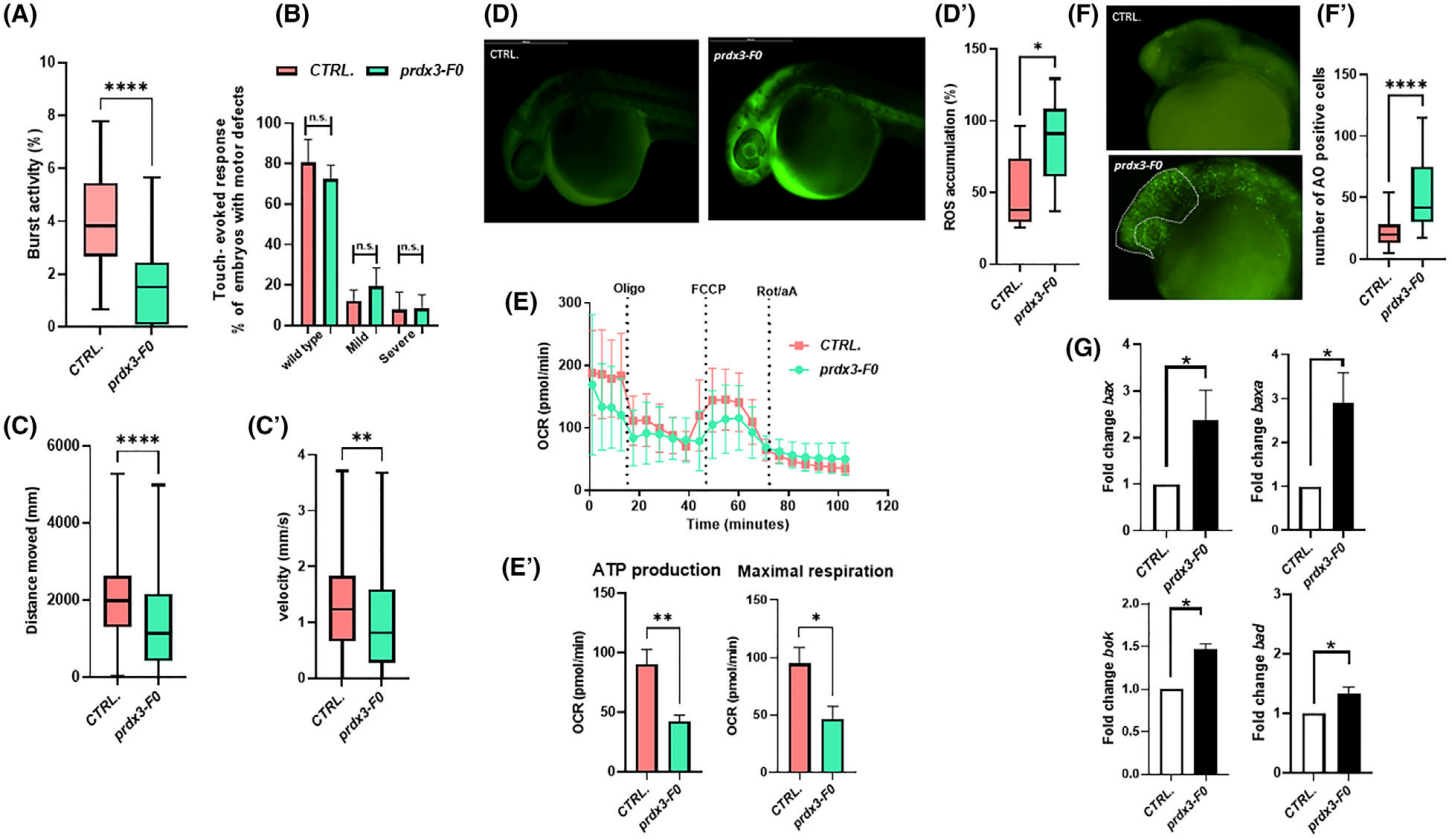
(A) The tail‐coiling test results showed a significant decrease in burst activity in *prdx3‐*F0 animals compared with controls (*N* = 50 per group) (****p ≤ 0.0001, statistics were calculated using the Mann–Whitney *U* test). (B) Touch‐evoked response test analysis showed slightly decreased movement in response to touch stimulus in morphants at 48 hpf compared with controls (statistics were calculated using chi‐square tests). (C and C′) Automated analysis of spontaneous motor activity revealed a reduction in swim distance and velocity in mutant F0 larvae at 120 hpf compared with control siblings (*N* = 200 per group in three independent experiments) (****p < 0.001 and **p < 0.01, statistics were calculated using the Mann–Whitney *U* test). (D) Representative fluorescence images of ROS generation in zebrafish larvae at 30 hpf (*N* = 30 per group). (D′) Graphs show the quantitative analysis of fluorescent signals. (*p < 0.05, statistics were calculated using the Mann–Whitney *U* test). (E and E′) Mitochondrial respiratory analysis of controls (*N* = 20) and mutant F0 larvae (*N* = 20) at 120 hpf (*p < 0.05 and **p < 0.01, statistics were calculated using the Mann–Whitney *U* test). (F and F′) Representative fluorescence images showing the presence, visible to the naked eye, of AO‐positive cells in controls and mutant F0 embryos at 30 hpf (lateral views). AO‐positive cells were counted in the area defined by the yellow line (****p < 0.001, statistics were calculated using the Mann–Whitney *U* test). (G) qRT‐PCR analysis revealed increases in apoptosis‐related gene expression, once the mRNA expression levels had been normalized to β‐Actin. Three independent RNA samples from controls and *prdx3‐*F0 larvae at 120 hpf were analyzed (*p ≤ 0.05, statistics were calculated using Student's *t*‐test). AO, acridine orange; *N*, number of evaluated embryos in total; n.s., not significant; OCR, oxygen consumption rate; ROS, reactive oxygen species. Error bars indicate standard error of the mean.

Thus, we characterized the *prdx3* “crispant” mutant F0 line (Fig. S1) and observed no toxic effects or morphological defects at 48 hpf (Fig. S1). Due to the lack of a reliable specific antibody for zebrafish, it was not possible to test protein abundance in depleted embryos through western blotting. However, qRT‐PCR analysis showed a significant decrease in the level of zebrafish *prdx3* transcript (Fig. S1). Analysis of tail flicks at 30 hpf in mutant F0 embryos revealed a significant decrease in burst activity (i.e., the percentage of time an embryo is moving) compared with controls (Fig. 2A). Touch‐evoked response assay at 48 hpf showed a slight reduction in responses to tail tap in F0 embryos compared with control siblings (Fig. 2B). At 120 hpf, video tracking data revealed significantly reduced locomotor activity in F0 larvae compared with controls, in terms of both velocity and distance covered (Fig. 2C,C′). Fluorescence measurements in mutant zebrafish F0 embryos showed a significant increase in susceptibility to oxidative stress (Fig. 2D,D′), and oxygen consumption rate studies revealed impaired mitochondrial bioenergetics in 120‐hpf larvae compared with controls, with significant reductions in ATP production and maximal respiration (Fig. 2E,E′). As already seen in cerebellar medulloblastoma cells,^11^ we observed a likely ROS‐triggered increment of acridine orange‐positive cells (Fig. 2F,F′), as confirmed by the significant increase in transcription levels of typical apoptosis‐associated genes^32^ (Fig. 2G).

## Discussion

In this work, we expanded the allelic heterogeneity associated with *PRDX3* pathogenic variants and highlighted the role of *prdx3* in zebrafish development. PRDX3 is a mitochondrial peroxiredoxin that plays a crucial role in energy metabolism by detoxifying ROS.^7^ Several neurological disorders, including different forms of ataxia, are associated with increased levels of free radicals^33^, ^34^ since the central nervous system, given its high oxygen demand, is particularly vulnerable to oxidative stress.^35^ ROS are signaling molecules important in maintaining normal tissue homeostasis.^36^ However, when intracellular ROS concentrations are not tightly controlled and exceed the cellular antioxidant capacity, they can damage all macromolecules, including lipids, proteins, and nucleic acids, leading to defects in their physiological functions.^37^, ^38^ For this reason, considerable attention has been paid to the role of deregulated expression and activity of antioxidant enzymes in neurodegeneration,^7^ and many studies have been conducted to validate the therapeutic roles of antioxidants in ARCA. Biallelic mutations in *PRDX3* have recently been reported to cause SCAR32,^11^, ^12^, ^13^, ^39^ a rare form of ARCA to date reported in only 15 patients whose clinical features and MRI findings were similar to those of our patient. Our case supports the evidence that oculomotor abnormalities occur in SCAR32, and represent, together with cerebellar ataxia, a possible red flag of the condition. To analyze the effect of stable downregulation of *prdx3* during neurodevelopment, we developed a new “crispant” *prdx3* zebrafish model that showed locomotor deficits, higher ROS production, and bioenergetic defects probably due to alterations in mitochondrial functionality. Altering mitochondrial membrane permeability and structure, membrane potential, and the respiratory chain has the effect of altering apoptosis in a caspase‐dependent manner.^40^ Although apoptosis occurs naturally during neural development,^41^ we observed that mutant “crispant” *prdx3* embryos displayed an increase in cell death, particularly in the anterior region of the central nervous system. As reported in the *prdx3*‐knockdown fruit fly model, which shows a decreased lifespan, we found that F0 mutant larvae showed increased susceptibility to ROS‐triggered apoptosis^11^ and significantly enhanced expression of several apoptosis‐associated genes. All these findings suggest that the endogenous apoptotic signaling pathway is activated by loss of *prdx3*‐induced oxidative stress in zebrafish larvae. Overall, our study provides novel insights into the consequences of impaired *PRDX3*. The novel *prdx3*‐deficient zebrafish model could help to pave the way for future drug discovery and optimization in mammalian models.

## Conclusions

In conclusion, a novel mutation in *PRDX3* is here reported as the genetic cause of SCAR32, a disorder whose red flags seem to include cerebellar atrophy, delayed neurodevelopment, and impaired oculomotor function. In addition, we generated a novel vertebrate (zebrafish) model to investigate the consequences of *prdx3* depletion on neurodevelopment and thus provided a new tool for investigating, in vivo, the early stages of locomotor impairment. We believe that *prdx3*‐deficient zebrafish embryos might offer a suitable platform for in vivo drug discovery studies and therefore allow preliminary testing of new treatment avenues ahead of more costly studies in mice.

## Conflict of Interest

The authors declare no competing interests.

## Author Contributions

All authors contributed to the study conception and design. Material preparation, data collection, and analysis were performed by Valentina Naef, Martina Troisi, Maria Lieto, Rosa Di Micco, Flavia Privitera, Alessandro Tessitore, Alessandro Filla, Sara Satolli, Rosa Pasquariello, and Stefano Doccini. The first draft of the manuscript was written by Valentina Naef and Filippo M. Santorelli; all authors commented on previous versions of it. All the authors read and approved the final manuscript. All authors attest that they meet the current ICMJE criteria for authorship.

## Funding Information

This work was partially supported by the Italian Ministry of Health (the EJP‐RD network PROSPAX), by Ricerca Finalizzata RF‐2019‐ 12370417, and supported in part by Ricerca Corrente 2023 (to FMS and SD).

## Supporting information


Appendix S1.

Figure S1.

Figure S2.

Captions.
