# Supplementary material for: SCAR32: Functional characterization and expansion of the clinical‐genetic spectrum
Source: Ann Clin Transl Neurol. 2024 Jun 5;11(7):1879–86. doi: 10.1002/acn3.52094 (PMC11251466; doi:10.1002/acn3.52094)
Supplement: Supplementary file 1 — Appendix S1. [file ACN3-11-1879-s001.docx]

**Supplementary methods**

***Oxygen consumption rate measurements***

Oxygen consumption rate (OCR) was measured in skin fibroblasts using an XFe24 analyzer (Seahorse Bioscience, Agilent, Santa Clara, CA). Cells were plated in XF 24-well cell culture microplates at a density of 5E + 04 cells/well. Measurements of endogenous respiration were performed with non-buffered DMEM medium supplemented with 1 mM pyruvate, 2 mM glutamine, and 10 mM glucose. After baseline measurements, OCR was analyzed by the sequential injection of 1 mM of oligomycin, 2 mM of carbonyl-cyanide 4-(trifluoromethoxy) phenylhydrazone, and 0.5 mM of rotenone plus antimycin A (all chemicals were from Sigma Aldrich, St. Louis, MO). Hoechst 33342 was included in the last injection in the XF analysis protocol for data normalization. Fluorescence was measured post-assay, as a function of the number of cells.

***Determination of reactive oxygen species***

For the evaluation of intracellular reactive oxygen species (ROS) production, skin fibroblasts were labeled with 25 μM of *in vivo* oxidative stress marker 2′,7′-dichloro-dihydrofluorescein diacetate (H2DCFDA) (#8206004, Abcam, Cambridge, MA, USA) for 45 min at 37 °C, and then cultured for an additional hour in the presence/absence of hydrogen peroxide at 500 nM to mimic ROS stimulus. Cells were then analyzed on a SpectraMax® ID3 plate reader (Molecular Devices, San Jose, CA) at a wavelengths Ex/Em: 485/535 nm, and ROS levels between treated and untreated condition were expressed as relative fluorescent units after background subtraction, and normalized to Hoechst 33342 intensity, as a function of the number of cells.
