# Supplementary figures and images for: SCAR32: Functional characterization and expansion of the clinical‐genetic spectrum
Source: Ann Clin Transl Neurol. 2024 Jun 5;11(7):1879–86. doi: 10.1002/acn3.52094 (PMC11251466; doi:10.1002/acn3.52094)

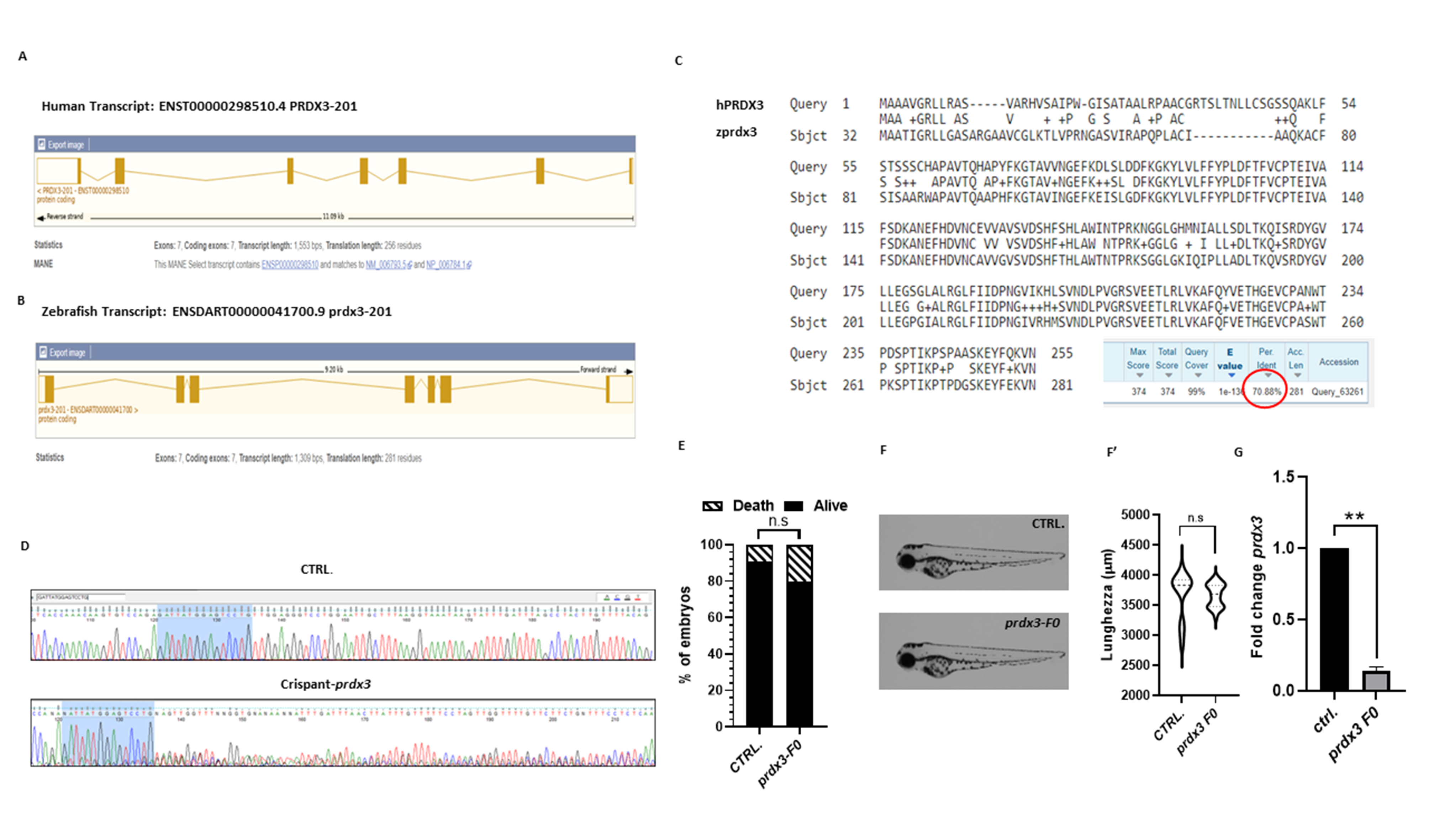

Supplement: Supplementary file 2 — Figure S1. [file ACN3-11-1879-s003.tif]

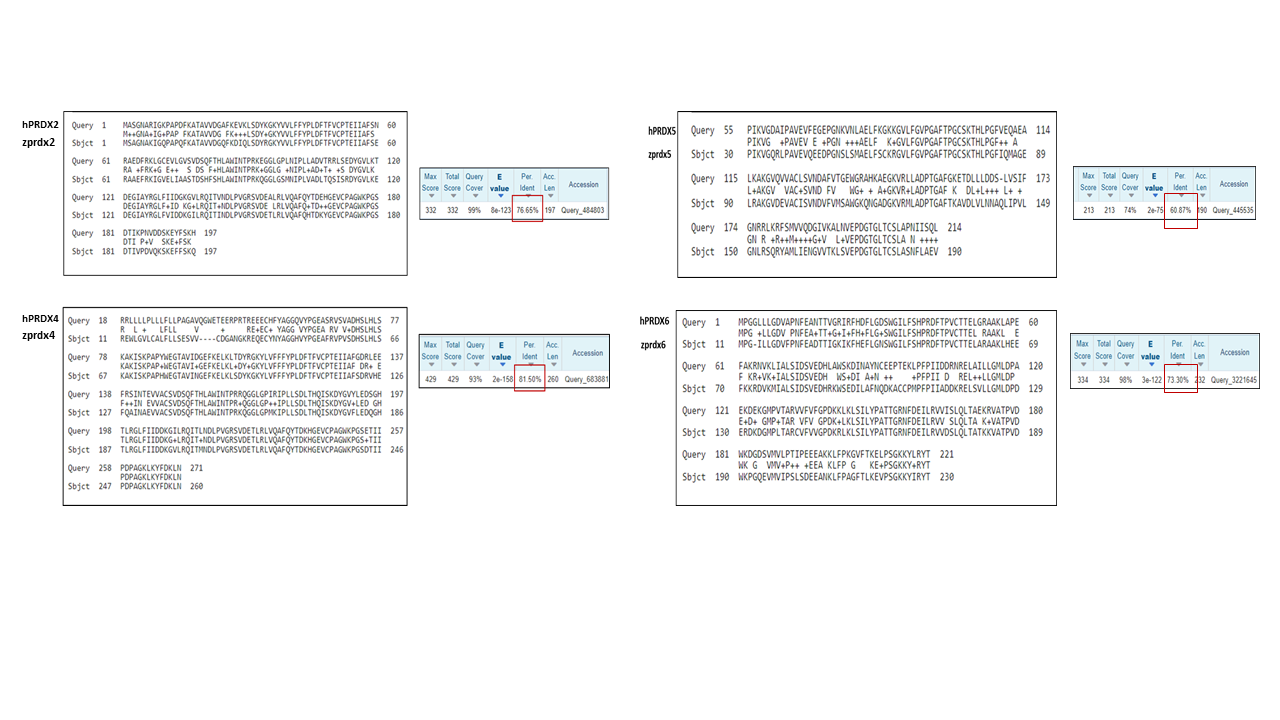

Supplement: Supplementary file 3 — Figure S2. [file ACN3-11-1879-s004.tif]
