# Supplementary material for: SCAR32: Functional characterization and expansion of the clinical‐genetic spectrum
Source: Ann Clin Transl Neurol. 2024 Jun 5;11(7):1879–86. doi: 10.1002/acn3.52094 (PMC11251466; doi:10.1002/acn3.52094)
Supplement: Supplementary file 4 — Captions. [file ACN3-11-1879-s002.docx]

**Appendix:** Cellular study methods

**Supplementary Figure 1**. Generation of *prdx3* “crispant” zebrafish. (A) Gene structure of *PRDX3* in *Homo sapiens* and (B) *Danio Rerio*. (C) *In silico* analysis of the amino acid sequence of prdx3 in *Danio rerio* and in *Homo sapiens* suggests that prdx3 is the fish ortholog of the human protein. (D) Sequencing chromatographs of control and mutant F0 specimens and alleles. The region highlighted in the chromatogram is the sgRNA targeting sequence that directed Cas9 nuclease activity. Sanger sequencing confirmed the strategy CRISPR/Cas9 editing efficacy used in this work.(E) Rate of embryo survival. (F) Lateral-view photographs of representative control and mutant F0 larvae. (F’) No dysmorphology and full length was noted at 120 hpf. (G) qRT-PCR analysis revealed a decrease in the level of *prdx3* mRNA expression, normalized to β-actin. Three independent RNA samples from *prdx3*-F0 larvae at 120 hpf and from controls were analyzed. ** p ≤ 0.01, calculated using Student’s t-test. The values are expressed as mean ± standard deviation (SD). Abbreviations: n, total number of evaluated embryos; error bars indicate standard errors of the means; n.s., not significant.

**Supplementary Figure 2.** Multiple sequence alignments of prdx amino acid sequences in *Danio rerio* and in *Homo sapiens* revealed high % of identity for all prdx protein family members among fish and human, indicating that peroxiredoxins are highly evolutionary conserved across different species.
